# *Toxoplasma gondii* infection in meat animals from Africa: Systematic review and meta-analysis of sero-epidemiological studies

**DOI:** 10.14202/vetworld.2017.194-208

**Published:** 2017-02-16

**Authors:** Aretas Babatoundé Nounnagnon Tonouhewa, Yao Akpo, Philippe Sessou, Camus Adoligbe, Eric Yessinou, Yaovi Gildas Hounmanou, Marc Napoléon Assogba, Issaka Youssao, Souaïbou Farougou

**Affiliations:** 1 Laboratory of Research in Applied Biology, Polytechnic School of Abomey-Calavi, University of Abomey-Calavi, 01 P.O. Box 2009, Cotonou, Benin; 2 Laboratory of Ecology, Health and animal Production, Faculty of Agronomy, University of Parakou, P.O. Box 123 Parakou, Benin; 3 Department of Veterinary Medicine and Public Health, Sokoine University of Agriculture, P.O. Box 3121, Chuo Kikoo, Morogoro, Tanzania

**Keywords:** animal health, meta-analysis, *Toxoplasmosis*, zoonosis

## Abstract

**Aim:**

*Toxoplasma gondii* is an ubiquitous apicomplexan parasite which causes toxoplasmosis in humans and animals. Felids especially cats are definitive hosts and almost all warm-blooded mammals, including livestock and human can serve as intermediate hosts. Food animals can be reservoirs for *T. gondii* and act as one of the sources for parasite transmission to humans. The objective of this study is to collect serological data on the prevalence of anti-*T. gondii* antibody, and risk factors for certain food animals from Africa to provide a quantitative estimate of *T. gondii* infection among these species from different African countries.

**Materials and Methods:**

Four databases were used to search seroepidemiological data on the prevalence of anti-*T. gondii* antibody in food animals between 1969 and 2016 from African countries. The search focused on data obtained by serologic test in food animals and meta-analyses were performed per species.

**Results:**

A total of 30,742 individual samples from 24 countries, described in 68 articles were studied. The overall estimated prevalence for toxoplasmosis in chicken, camel, cattle, sheep, goat, pig were 37.4% (29.2-46.0%), 36% (18-56%), 12% (8-17%), 26.1% (17.0-37.0%), 22.9% (12.3-36.0%), and 26.0% (20-32.0%), respectively. Moreover, major risk factor of infection was age, farming system, and farm location.

**Conclusions:**

A significant variation in the seroepidemiological data was observed within each species and country. The results can aid in an updated epidemiological analysis but also can be used as an important input in quantitative microbial risk assessment models. Further studies are required for a better and continual evaluation of the occurrence of this zoonotic infection.

## Introduction

*Toxoplasma gondii* is a coccidian parasite that is globally widespread and causes a common infection in animal and human. The parasite was described for the first time in a North African rodent (*Ctenodactylus gondii*) independently by Nicolle, Manceaux, and Splendore in 1908 [1]. Felids especially cats are definitive hosts and represent the key element in the epidemiology of disease caused by this parasite. Almost all warm-blooded mammals, including livestock, and human can serve as intermediate hosts [2]. *T. gondii* can infect all homeotherms and is responsible for many abortions and fetal malformations in human and animal [3].

According to estimates, approximately 1/3 of the world’s population would be infected [4] and *T. gondii* infection represent the most prevalent parasitic zoonotic disease worldwide [5]. This parasite is present on all continents, and the rate of infection vary highly according to areas [2]. However, climate change has led to an increase of T. gondii infections in different regions of the world as a result of changing environmental conditions [6].

Humans get infected after ingesting undercooked or raw meat, by ingesting cat-shed oocysts via contaminated soil, food, water or congenitally by transplacental transmission of tachyzoites [5]. However, the clinical disease is seen only in few cases with serious consequences in immunocompromised people and pregnant women [7]. Toxoplasmosis is a major cause of reproductive failure in sheep, goats, and pigs [8,9] and also recognized as a serious problem in immunocompromised patients particularly AIDS patient [10,11]. Furthermore, recent studies have shown that toxoplasmosis is a risk factor for schizophrenia [12], epilepsy [13], and traffic accidents [14] and highly virulent atypical strains of *T. gondii* have been incriminated with pneumonia, even in immunocompetent people [15].

Toxoplasmosis, especially cerebral toxoplasmosis has become the most common opportunistic infection of the central nervous system during HIV infection in the world [10,11]. Africa is the most continent affected by HIV/AIDS infection that affects about 30 million people on the continent [16]. Unluckily countries most affected are those least able to meet the cost of prevention and treatment of disease. Thus, toxoplasmosis has become an important public health problem on the continent account to the severity of the infection in AIDS patients more frequent in Africa. The absence of public health schemes to manage the spread of this disease places African populations at risk of ongoing and possibly increasing incidence and prevalence, as well as a corresponding increase in mortality and morbidity due to toxoplasmosis [17].

Food animals are important livestock species, especially in developing countries and their products (meat and milk) are used in various parts of the world. Pork and chicken are the most consumed meat in the world with global production estimated at 115.5 and 108.7 million tons in 2014 [18]. In Africa; cattle, chicken, sheep, goat, pig, and camel represent the most consumed animal species. According to estimate, the meat production on the continent was estimated at 17352 thousands of tons in 2013 and increasing every year [18]. Food animals can be reservoirs for *T. gondii* and act as one of the sources for parasite transmission to humans. Many epidemiologic studies have found an association between consumption of undercooked or raw meat and *T. gondii* infection in human [19,20]. Based on limited population-based data, the Food and Agriculture Organization and World Health Organization estimated that approximately 22% of human *T. gondii* infections are meatborne [21].

To detect *T. gondii* in meat animal, three methods have been used. These methods include serological assays, bioassay, and polymerase chain reaction (PCR) [22]. Among these three methods, serological assays are rapid and have good accuracy for detecting anti-*T. gondii* antibodies in food animals [23-25] and the modified agglutination test (MAT) and enzyme-linked immunosorbent assay (ELISA), are the most commonly used serological test.

Compared to other continents, few studies have been conducted on toxoplasmosis in Africa. Studies available on the seroprevalence of toxoplasmosis in African countries are still fragmented, except some countries including Ethiopia where the infection is well documented. Therefore, there have been a few studies on seroprevalence rates of *T. gondii* in animal species on the continent, and the results of the available studies are sometimes contradictory.

Meta-analysis is a method to synthesize the results of various studies for a given question and was applied to a wide range of food safety questions [26]. The quantitative results obtained from meta-analysis were used as inputs in risk assessment models [27]. According to Gliner *al*. [28], the advantages of performing a meta-analysis include providing summary statistics based on multiple individual studies, increasing precision in estimating effects, and taking the size of studies into account.

The aim of this systematic review and meta-analysis study is to collect serological data on the prevalence of anti-*T. gondii* antibody, and risk factors for most consumed food animals from Africa to provide a quantitative estimate of *T. gondii* infection among these species.

## Materials and Methods

### Ethical approval

This study did not require an ethical approval as it was based on information/data retrieved from published studies already available in the public domain.

### Data sources and searches

We conducted a systematic literature review on the seroprevalence of *T. gondii* among food animals in African countries as per preferred reporting items for systematic reviews and meta-analyses criteria [29]. Relevant studies were identified by searching four literature databases including PubMed, Web of Science, Scopus, and Google Scholar. No time limitation was imposed. The search criteria were specified in advance and the search was executed on 11/12/2015 and last updated on 01/04/2016. The search string used was the following: “toxoplasma” OR “toxoplasmosis” AND “seroprevalence” OR “seroepidemiology” AND “sheep” OR “goat” OR “pig” OR “cattle” OR “chicken” OR “camel” AND “Africa”.

### Data collection and eligibility criteria

For this review, only articles written in English and French were considered. Two investigators studied titles and abstract of all the articles and retrieved data. Several criteria were used to select eligible studies (1) study were performed in animals raised in different African countries; (2) the prevalence of *T. gondii* had to be detected by serologic methods (ELISA, MAT, direct agglutination test [DAT], modified direct agglutination test [MDAT], indirect fluorescent antibody test [IFAT], latex agglutination test [LAT], and Sabin and Feldman test [SFT]); (3) samples had to originate from food animals (cattle, chicken, camel, pigs, sheep and goat); (4) samples had to be collected from animals which were naturally infected; (5) sampling strategy had to be directed toward a random population; (6) the sample size was <35. The extracted data included: Year of publication, host, country of the study, sample size, number of cases, diagnostic test, and risk factors. Reference lists of full-text publications and textbooks were also examined to identify studies not retrieved by the original search. All studies were coded according to the previously chosen parameters, and data were recorded in Microsoft Excel table.

### Quality and bias assessment of eligible studies

Each eligible study was assessed for quality and bias using the risk of bias tool, which is a methodological quality assessment checklist for prevalence studies [30]. 10 questions were contained in this checklist, and each of the 10 questions was scored 1 or 0 based on the quality of each eligible study [30]. This questions were as follows:

Q1: Was the study’s target population a close representation of the national population in relation to relevant variables?
Q2: Was the sampling frame a true or close representation of the target population?
Q3: Was some form of random selection used to select the samples, or, was a census undertaken?
Q4: Was the likelihood of non-response bias minimal?
Q5: Were data collected directly from the subjects (as opposed to a proxy)?
Q6: Was an acceptable case definition used in the study?
Q7: Was the study instrument that measured the parameter of interest shown to have reliability and validity (if necessary)?
  Yes (if using MAT, ELISA, DAT, and MDAT),
  No (using other serologic detection methods).
Q8: Was the same mode of data collection used for all subjects?
Q9: Was the length of the shortest prevalence period for the parameter of interest appropriate? Q10: Were the numerator(s) and denominator(s) for the parameter of interest appropriate?

Eight different detection methods were used in these eligible studies. For question 7, which was to determine the reliability and validity of the measurement, MAT, ELISA, DAT and MDAT were considered as reliable diagnostic methods (score 1) [24,25], and other diagnostic tests such as LAT, indirect immunoflourescent assay (IFA), indirect hemagglutination assay (IHA), SFT, were determined as unreliable methods (score 0). A quality score was determined by rescaling the sum of scores of each eligible study between 0 and 1 [30]. Quality assessment was completed independently by two assessors, and a table of quality score computation for each eligible study is provided in the Supplementary Table-S1.

**Supplementary Table-S1:** Quality score assessment based on the “risk of bias tool“ (Hoy *et al*., 2012).

Species	Study	Q1	Q2	Q3	Q4	Q5	Q6	Q7	Q8	Q9	Q10	Summary
Pig	Bamba *et al*. [36]	0	1	1	1	1	1	1	1	1	1	9
Chicken	Gebremedhin *et al*. [37]	0	1	1	1	1	1	1	1	1	1	9
Goat	Abdel-Hafeez *et al*. [38]	0	0	1	1	1	1	0	1	1	1	7
Sheep	Dechicha *et al*. [39]	0	0	0	1	1	1	0	1	1	1	6
Goat	Dechicha *et al*. [39]	0	0	0	1	1	1	0	1	1	1	6
Cattle	Dechicha *et al*. [39]	0	0	0	1	1	1	0	1	1	1	6
Cattle	Onyiche and Ademola [40]	0	0	1	1	1	1	1	1	1	1	8
Pig	Onyiche and Ademola [40]	0	0	1	1	1	1	1	1	1	1	8
Cattle	Elfahal *et al*. [41]	0	0	0	0	1	1	1	1	1	1	6
Pig	Gebremedhin *et al*. [42]	0	0	1	1	1	1	1	1	1	1	8
Camel	Hadush *et al*. [43]	0	1	1	1	1	1	1	1	1	1	9
Sheep	Lahmar *et al*. [44]	0	0	0	1	1	1	1	1	1	1	7
Sheep	Hammond-Aryee *et al*. [45]	0	1	1	1	1	1	1	1	1	1	9
Chicken	Boughattas *et al*. [46]	0	0	1	1	1	1	1	1	1	1	8
Chicken	Ayinmode and Olaosebikan [47]	0	1	1	1	1	1	1	1	1	1	9
Goat	Davoust *et al*. [48]	0	0	1	1	1	1	1	1	1	1	8
Sheep	Davoust *et al*. [48]	0	0	1	1	1	1	1	1	1	1	8
Sheep	Gebremedhin and Gizaw [49]	0	1	1	1	1	1	1	1	1	1	9
Goat	Gebremedhin and Gizaw [49]	0	1	1	1	1	1	1	1	1	1	9
Sheep	Gebremedhin *et al*. [50]	0	1	1	1	1	1	1	1	1	1	9
Goat	Gebremedhin *et al*. [50]	0	1	1	1	1	1	1	1	1	1	9
Cattle	Medani and Kamil [51]	0	0	0	1	1	1	1	1	1	1	7
Sheep	Medani and Kamil [51]	0	0	0	1	1	1	1	1	1	1	7
Camel	Kadle [52]	0	1	1	1	1	0	1	1	1	7
Camel	Gebremedhin *et al*. [53]	0	1	1	1	1	1	1	1	1	1	9
Chicken	Tilahun *et al*. [54]	0	1	1	1	1	1	1	1	1	1	9
Chicken	Aboelhadid *et al*. [55]	0	0	1	1	1	1	1	1	1	1	8
Goat	Zwedu *et al*. [56]	0	1	1	1	1	1	1	1	1	1	9
Goat	Swai and Kaaya [57]	0	1	1	1	1	1	0	1	1	1	8
Cattle	Ndou *et al*. [58]	0	1	1	1	1	1	1	0	1	1	8
Pig	Ayinmode and Olaosebikan [59]	0	1	1	1	1	1	1	1	1	1	9
Sheep	Gebremedhin *et al*. [60]	0	1	1	1	1	1	1	1	1	1	9
Sheep	Bamba *et al*. [61]	0	0	1	1	1	1	1	1	1	1	8
Sheep	Al-mabruk *et al*. [62]	1	1	1	1	1	1	0	1	1	1	9
Sheep	Gharbi *et al*. [63]	0	0	1	1	1	1	1	1	1	1	8
Chicken	Barakat *et al*. [64]	0	0	1	1	1	1	1	1	1	1	8
Pig	Rakotoharinome *et al*. [65]	0	0	1	1	1	1	1	1	1	1	8
Cattle	Swai and Schoonman [66]	0	0	0	1	1	1	0	1	1	1	6
Camel	Khalil and Abdel Gadir [67]	0	1	1	1	1	1	0	1	1	1	8
Sheep	Khalil and Abdel Gadir [67]	0	1	1	1	1	1	0	1	1	1	8
Sheep	Boughattas and Bouratbine [68]	0	1	1	1	1	1	1	1	1	1	9
Sheep	Kamani *et al*. [69]	0	0	1	1	1	1	1	1	1	1	8
Goat	Kamani *et al*. [69]	0	0	1	1	1	1	1	1	1	1	8
Cattle	Ibrahim *et al*. [70]	0	0	0	1	0	1	0	1	1	1	5
Chicken	Dubey *et al*., [71]	0	0	0	1	1	1	1	1	1	1	7
Chicken	Lindstrom *et al*. [72]	0	0	1	1	1	1	1	1	1	1	8
Sheep	Shapaan *et al*. [73]	0	0	0	1	1	1	1	1	1	1	7
Goat	Teshale and Dumaitre [74]	0	1	1	1	1	1	1	1	1	1	9
Sheep	Samra *et al*. [75]	0	1	1	1	1	1	1	1	1	1	9
Chicken	Dubey *et al*. [76]	0	0	1	1	1	1	1	1	1	1	8
Chicken	Deyab and Hassanein [77]	0	1	1	1	1	1	1	1	1	1	9
Goat	Hove *et al*. [78]	1	1	1	1	1	1	0	1	1	1	9
Cattle	Schoonman *et al*. [79]	0	1	1	1	1	1	0	1	1	1	8
Pig	Hove *et al*. [80]	0	1	1	1	1	1	0	1	1	1	8
Sheep	Sawadogo *et al*. [81]	0	1	1	1	1	1	1	1	1	1	9
Sheep	Negash and Tilahun [82]	0	1	1	1	1	1	1	1	1	1	9
Goat	Negash and Tilahun [82]	0	1	1	1	1	1	1	1	1	1	9
Chicken	Dubey *et al*. [83]	0	0	0	1	1	1	1	1	1	1	7
Cattle	Joshua and Akinwumi [84]	0	1	1	1	1	1	0	1	1	1	8
Chicken	El-Massry *et al*. [85]	0	0	1	1	1	1	1	1	1	1	8
Sheep	Van der Puije *et al*. [86]	1	1	1	1	1	1	1	1	1	1	10
Goat	Van der Puije *et al*. [86]	1	1	1	1	1	1	1	1	1	1	10
Goat	Bisson *et al*. [87]	1	1	1	1	1	1	1	1	1	1	10
Pig	Arkoh Mensah *et al*. [88]	1	1	1	1	1	1	1	1	1	1	10
Pig	Hove and Dubey [89]	0	0	0	1	1	1	1	1	1	1	7
Camel	Hilali *et al*. [90]	0	1	1	1	1	1	1	1	1	1	9
Chicken	Hassanain and Elfadaly [91]	0	0	1	1	1	1	0	1	1	1	7
Sheep	Deconinck *et al*. [92]	0	0	0	1	1	1	0	1	1	1	6
Sheep	Achu-Kwi and Ekue [93]	0	0	1	1	1	1	0	1	1	1	7
Sheep	El-Ghaysh and Mansour [94]	0	0	1	1	1	1	1	1	1	1	8
Goat	Amin and Silsmore [95]	0	1	1	1	1	1	0	1	1	1	8
Sheep	Pangui *et al*. [96]	0	0	1	1	1	1	0	1	1	1	7
Camel	Elamin *et al*. [97]	0	1	1	1	1	1	0	1	1	1	8
Sheep	Pandley and Mansour [98]	1	1	1	1	1	1	1	1	1	1	10
Sheep	Weitzman *et al*. [99]	0	1	1	1	1	1	0	1	1	1	8
Sheep	Bekele and Kasali [100]	0	1	1	1	1	1	0	1	1	1	8
Goat	Bekele and Kasali [100]	0	1	1	1	1	1	0	1	1	1	8
Cattle	Bekele and Kasali [100]	0	1	1	1	1	1	0	1	1	1	8
Chicken	Aganga and Belino [101]	0	0	1	1	1	1	0	1	1	1	7
Goat	Falade [102]	0	0	1	1	1	1	0	1	1	1	7
Chicken	Rifaat *et al*. [103]	0	0	1	1	1	1	0	1	1	1	7

### Data analysis

Data were recorded in Microsoft Excel spreadsheet and analysed by MetaXL version 4.0 software (EpiGear Int Pty Ltd., Wilston) [31] for the meta-analyses and graphed as forest plot. For pooled prevalence analysis, random effects model was adopted over fixed effect model because there is more robust when analyzing heterogeneous studies [32]. Data were transformed by a double arcsine transformation as described by Barendregt *et al*. [33] to stabilize the variance. Publication bias was assessed by funnel plots representing the double arcsine transformation of the prevalence against the standard error [34]. Heterogeneity among studies was evaluated by Cochrane Q and I^2^ statistical methods. A significant value (p<0.05) in the Cochrane Q method suggests a real effect difference in the meta-analysis. A value of I^2^ was used to measure the inconsistency across studies. Values of 25%, 50%, and 75% were considered as having a low, moderate, and high degree of heterogeneity, respectively [35].

## Results

**Figure-1:**
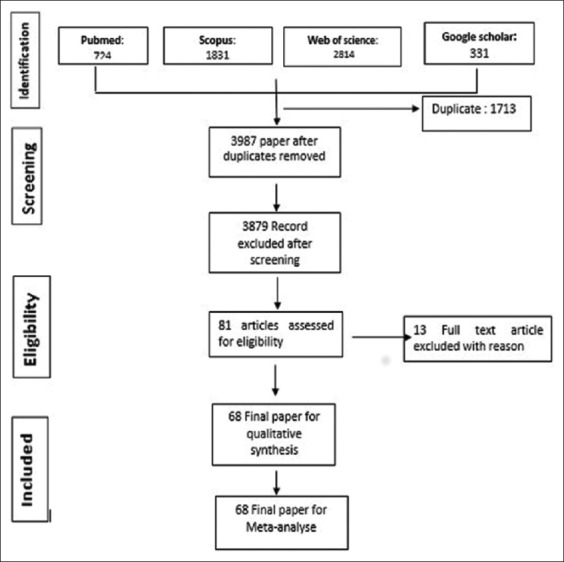
Schematic flow diagram describing the selection of relevant studies.

Schematic flow diagram describing the selection of relevant studies Figure-1.

### Characteristics of eligible studies

Figure-1 shows the flow diagram of the selection of eligible studies. A total of 5700 papers published between 1969 and 2016 were identified by literature search among the four database searched. After duplicate removed and irrelevant studies based on titles and abstracts, 81 articles were retrieved for detailed full-text analysis. 13 were excluded due to the following reasons: Two were not available; the sample size was lower than 35 in four study; the diagnosis was established on the basis of other methods than serologic test in seven studies. Table-1 shows the characteristics of included studies [36-103]. Finally, a total of 68 articles from 24 countries were included in this systematic review and meta-analysis study. Approximately, 60% (41/68) of the studies were published within the last 10 decade (2007-2016) of the review period. The regional distribution of studies was west Africa (18), East Africa (17), North Africa (21), Southern Africa (8), and Central Africa (4). Our analysis included a totally 30,742 individual samples distributed as follows: 14,272 sheep, 6355 goats, 3366 cattle, 2798 chickens, 2080 pigs, and 1621 camels. Eight different types of diagnostic tests were employed to evaluate *T. gondii* infection. These diagnostic methods were MAT, ELISA, IHA, DAT, MDAT, IFA, LAT, and SFT. The most used diagnostic tests in 47 year surveys were ELISA and MAT in 24 and 20 studies, which was followed by LAT (14), IHA (13), DAT (6), IFA (6), MDAT (3) and SFT (1). Sensitivity and specificity of diagnostic test are described in Table-2 as reported in literature.

**Table-1:** Characteristics of included studies.

Study No	Country	Author	Year	Hosts	Method	Sample size	Positive (%)	Quality score
1	Burkina-Faso	Bamba *et al*. [36]	2016	Pig	MAT	300	87 (29)	8
2	Ethiopia	Gebremedhin *et al.* [37]	2015	Chicken	MAT	601	183 (30.50)	9
3	Egypt	Abdel-Hafeez *et al*. [38]	2015	Goat	IHAT	100	64 (64)	7
4	Algeria	Dechicha *et al*. [39]	2015	Sheep, Goat, Cattle	IFAT	714	59 (8.26)	6
5	Nigeria	Onyiche *et al.* [40]	2015	Cattle, Pig	ELISA	512	117 (22.85)	9
6	Sudan	Elfahal *et al*. [41]	2015	Cattle	ELISA	181	24 (13.30)	6
7	Ethiopia	Gebremedhin *et al*. [42]	2015	Pig	DAT	402	129 (32.10)	9
8	Ethiopia	Hadush *et al.* [43]	2015	Camel	DAT	384	262 (68.20)	9
9	Tunisia	Lahmar *et al*. [44]	2015	Sheep, Goat, Cattle	MAT	261	82 (36.78)	7
10	South-Africa	Hammond-Aryee *et al.* [45]	2015	Sheep	ELISA	292	23 (8.00)	9
11	Tunisia	Boughattas *et al*. [46]	2014	Chicken	MAT	40	40 (100)	8
12	Nigeria	Ayinmode *et al.* [47]	2014	Chicken	MAT	225	81 (40.40)	9
13	Senegal	Davoust *et al.* [48]	2014	Cattle, Goat, Horse, Sheep	MAT	419	148 (35.33)	8
14	Ethiopia	Gebremedhin and Gizaw [49]	2014	Sheep, Goat	ELISA	184	48 (26.08)	9
15	Ethiopia	Gebremedhin *et al*. [50]	2014	Sheep, Goat	DAT	628	50 (17.62)	9
16	Sudan	Medani and Kamil [51]	2014	Cattle, Sheep	ELISA	540	153 (28.33)	7
17	Somalia	Kadle [52]	2014	Camel	LAT	64	4 (6.3)	7
18	Ethiopia	Gebremedhin *et al*. [53]	2014	Camel	DAT	455	220 (49.62)	9
19	Ethiopia	Tilahun *et al*. [54]	2013	Chicken	MAT	64	41 (64.00)	9
20	Egypt	Aboelhadid *et al.* [55]	2013	Chicken	MAT	215	30 (13.95)	8
21	Ethiopia	Zwedu *et al.* [56]	2013	Goat	ELISA	927	183 (19.70)	9
22	Tanzania	Swai and Kaaya [57]	2013	Goat	LAT	337	65 (19.30)	8
23	South-Africa	Ndou *et al*. [58]	2013	Cattle	ELISA	178	37 (20.8)	8
24	Nigeria	Ayinmode and Olaosebikan [59]	2013	Pig	ELISA	100	25 (25)	8
25	Ethiopia	Gebremedhin *et al*. [60]	2013	Sheep	ELISA	1130	357 (31.59)	9
26	Burkina-Faso	Bamba *et al.* [61]	2013	Sheep	MAT	339	96 (28.3)	8
27	Libya	Al-Mabruk *et al.* [62]	2013	Sheep	LAT	5806	4120 (71.00)	9
28	Tunisia	Gharbi *et al*. [63]	2013	Sheep	ELISA	350	38 (10.85)	8
29	Egypt	Barakat *et al.* [64]	2012	Chicken	ELISA	125	48 (38.40)	8
30	Madagascar	Rakotoharinome *et al.* [65]	2012	Pig	ELISA	250	57 (22.80)	8
31	Tanzania	Swai and Schoonman [66]	2012	Cattle	LAT	51	06 (12.80)	6
32	Sudan	Khalil and Abdel Gadir [67]	2011	Cattle, Camel, Sheep	LAT	200	76 (38.00)	7
33	Tunisia	Boughattas and Bouratbine [68]	2011	Sheep	MAT	158	28 (17.70)	9
34	Nigeria	Kamani *et al*. [69]	2010	Sheep, Goat	ELISA	744	42 (5.45)	8
35	Egypt	Ibrahim *et al.* [70]	2009	Cattle	ELISA	93	10 (10.75)	5
36	Ghana	Dubey *et al.*, [71]	2008	Chicken	MAT	85	40 (47.00)	7
37	Uganda	Lindstrom *et al*. [72]	2008	Chicken	MAT	50	25 (50.00)	8
38	Egypt	Shapaan *et al.* [73]	2008	Sheep	MAT	300	131 (43.70)	7
39	Ethiopia	Teshale and Dumaitre [74]	2007	Goat	MDAT	641	480 (74.80)	9
40	South-Africa	Samra *et al*. [75]	2007	Sheep	ELISA	600	26 (4.30)	9
41	Egypt	Dubey *et al*. [76]	2003	Chicken	MAT	108	51 (47.20)	8
42	Egypt	Deyab and Hassanein [77]	2005	Chicken	MAT	150	28 (18.1)	9
43	Zimbabwe	Hove *et al*. [78]	2005	Goat	IFAT	312	214 (68.58)	9
44	Tanzania	Schoonman *et al*. [79]	2010	Cattle	LAT	665	24 (3.60)	8
45	Zimbabwe	Hove *et al*. [80]	2005	Pig	IFAT	238	47 (26.79)	8
46	Morocco	Sawadogo *et al*. [81]	2005	Sheep	ELISA	261	72 (27.60)	9
47	Ethiopia	Negash and Tilahun [82]	2004	Sheep, Goat	MDAT	174	79 (45.40)	9
48	RDC, Mali, Burkina-Faso and Kenya	Dubey *et al*. [83]	2005	Chicken	MAT	80	29 (36.25)	7
49	Nigeria	Joshua and Akinwumi [84]	2003	Cattle	LAT	586	99 (16.9)	8
50	Egypt	El-Massry *et al*. [85]	2000	Chicken	MAT	150	28 (18.70)	8
51	Ghana	Van der Puije *et al.* [86]	2000	Sheep, Goat	ELISA	1258	384 (30.52)	10
52	Uganda	Bisson *et al*. [87]	2000	Goat	ELISA	784	240 (31.00)	10
53	Ghana	Arkoh Mensah *et al*. [88]	2000	Pig	ELISA	641	260 (40.60)	10
54	Zimbabwe	Hove and Dubey [89]	1999	Pig	MAT	97	9 (09.30)	7
55	Egypt	Hilali *et al.* [90]	1998	Camel	DAT	166	29 (17.40)	9
56	Egypt	Hassanain and Elfadaly [91]	1997	Chicken	IHAT	600	200 (33.33)	7
57	Burkina-Faso, Ivory-Coast, Djiboutia, Ethiopia, Niger, Senegal	Deconinck *et al*. [92]	1996	Sheep	IHAT	1042	15 (23.00)	6
58	Cameroon	Achu-Kwi and Ekue [93]	1994	Sheep	LAT	211	67 (31.80)	7
59	Egypt	El-Ghaysh and Mansour [94]	1994	Sheep	MAT	102	50 (49.00)	8
60	Nigeria	Amin and Silsmore [95]	1993	Sheep, Goat	LAT	465	37 (7.95)	7
61	Senegal	Pangui *et al.* [96]	1993	Sheep	IFAT	190	88 (46.30)	7
62	Sudan	Elamin *et al*. [97]	1992	Camel	LAT	482	323 (67.00)	7
63	Zimbabwe	Pandley and Van Knapen [98]	1992	Sheep	ELISA	216	13 (06.00)	10
64	Niger	Weitzman and Stem [99]	1991	Sheep	LAT	70	10 (14.00)	8
65	Ethiopia	Bekele and Kasali [100]	1989	Sheep, Goat, Cattle	IHAT	2437	349 (14.32)	8
66	Nigeria	Aganga and Belino [101]	1984	Chicken	IHAT	250	112 (44.80)	7
67	Nigeria	Falade [102]	1978	Goat	LAT	751	23 (3.06)	7
68	Egypt	Rifaat *et al*. [103]	1969	Chicken	DAT	85	17 (20.00)	7

MAT: Modified agglutination test, DAT: Direct agglutination test, MDAT: Modified direct agglutination test, ELISA: Enzyme-linked immunosorbent assay, LAT: Latex agglutination test, IFAT: Indirect fluorescent antibody test, IHAT: Indirect hemagglutination test

**Table-2:** Comparing diagnostic methods.

Diagnostic test	Study (%) N=68	Sensitivity (%)	Specificity (%)	References
MAT, DAT, MDAT	38.23	82.9	92.29	Dubey *et al*. [23]
ELISA	29.41	72.9	85.90	Dubey *et al*. [23]
LAT	17.64	45.9	96.90	Dubey *et al*. [23]
IHA	07.35	29.4	98.30	Dubey *et al.* [23]
IFA	05.88	80.40	91.40	Arthur and Blewett [103]
SFT	01.47	54,4	90,80	Dubey *et al*. [23]

MAT: Modified agglutination test, DAT: Direct agglutination test, MDAT: Modified direct agglutination test, ELISA: Enzyme-linked immunosorbent assay, LAT: Latex agglutination test, IHA: Indirect hemagglutination assay, IFA: Indirect immunoflourescent assay, SFT: Sabin and Feldman test

### Quality and bias assessments

**Figure-2:**
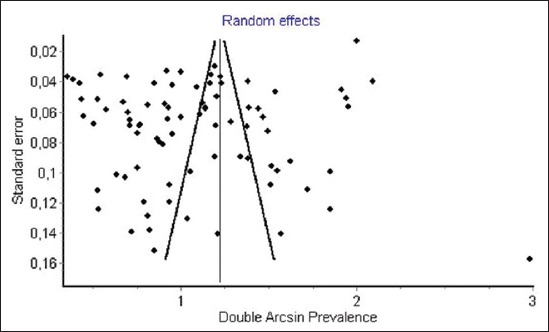
Funnel plot of double arcsinus seroprevalence estimates in food animals.

Supplementary Table-S1 (Appendix) represents the quality score of different eligible study. The quality score in 54/84 eligible studies ranged from 6 and 8 (Table-S1) [36-103]. It shows that the risk of bias in these studies was moderate. Besides, many of the eligible studies were conducted in regional and local farms or slaughterhouses, which were not representative of the national population of animals sampled in these countries. Only 5 of the 84 studies were conducted at the national level (Table-S1). Moreover, studies on animal toxoplasmosis were available only in 24 countries out of 54 of African continent. The risk of bias due to quality deficiency in eligible studies was mainly due to external validity criteria, while the flaws internal validity recorded in eligible studies concerned the use of diagnostic tests other than reference methods such as ELISA and MAT (Table-2) [104]. Finally, the symmetry in the funnel plots ruled out substantial publication bias (Figure-2).

### Population prevalence in food animals

#### Prevalence of anti-T. gondii antibody in sheep

**Figure-3:**
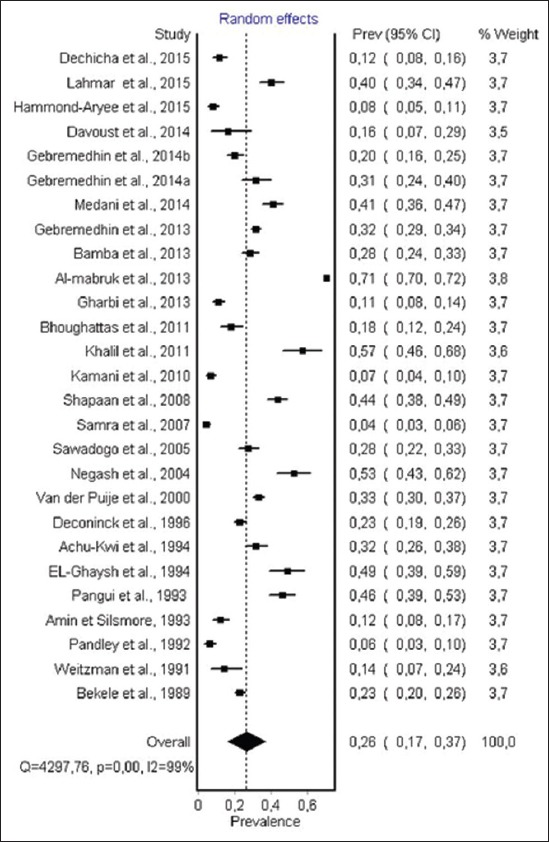
Forest plot of *Toxoplasma gondii* infection prevalence in sheep (random effect model). In a forest plot, each study is represented by a line, the width of the line represents the confidence intervals for effect estimate of each study, and area of the box indicates the weight given to each study. This description of forest plot is applied to all forest plots presented in Figures-3-8.

Data from 27 studies from 17 countries were obtained among sheep. 10 studies used ELISA, 6 studies used MAT, 5 used LAT, 2 used IHA and IFA, DAT and MDAT were used in 1 study, respectively. A total number of individual samples was 14,272. The prevalence of toxoplasmosis in sheep varied from 4.30% to 68.00%. The random effect model used in the meta-analysis (Figure-3) gave an overall estimated prevalence of 26.1% (95% confidence interval [CI] 17.0-37.0%). The result of heterogeneity was also 96.83% (95% CI 96.18-97.38%) for the degree of inconsistency.

#### Prevalence of anti-T. gondii antibody in goats

**Figure-4:**
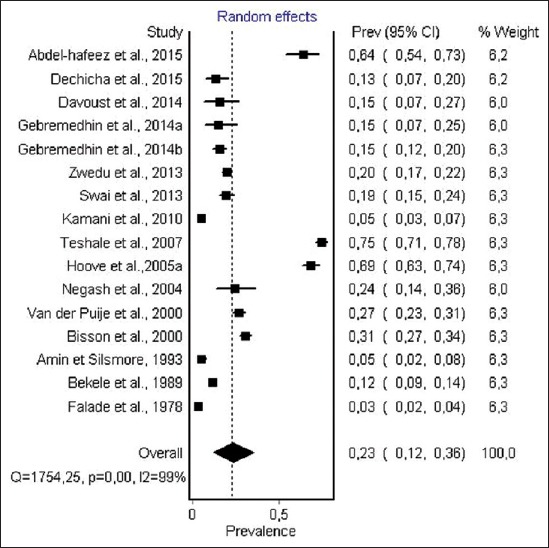
Forest plot of *Toxoplasma gondii* infection prevalence in goat (random-effects model).

The data obtained from *T. gondii* infection in goat result from 17 studies from 9 countries. The reported prevalence ranged from 3.6% to 74.8%. For diagnostic methods, 5 studies performing ELISA, 2 studies performing LAT, 2 studies, performing MDAT, IFAT, IHA, respectively, and 1 study performing MAT and DAT, respectively. The total number of individual samples was 6355. The random effect model (Figure-4) gave an overall estimated prevalence of 22.9% (95% CI 12.3-36.0%). The result of heterogeneity was also 99.1% (95% CI 99.0-99.3%) for the degree of inconsistency.

#### Prevalence of anti-T. gondii antibody in cattle

**Figure-5:**
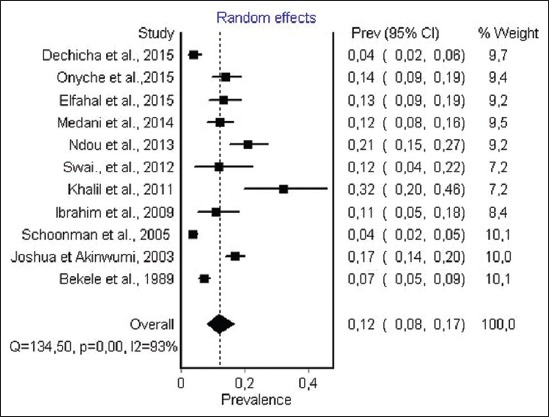
Forest plot of *Toxoplasma gondii* infection prevalence in cattle (random-effects model).

Information on *T. gondii* infection in cattle was obtained from 11 studies from 8 countries. 5 studies performing LAT; 4 studies performing ELISA; 4 studies performing IFAT and IHA, respectively. The total number of individual samples was 3366. *T. gondii* infection prevalence among cattle ranged from 3.6% to 32%. The random effect model (Figure-5) gave an overall estimated prevalence of 12% (95% CI 8-17%, p<0.001). The result of heterogeneity was also 92.56% (95% CI 88.65-95.12) for the degree of inconsistency. A detailed description of each study is given in Figure-5.

#### Prevalence of anti-T. gondii antibody in camels

**Figure-6:**
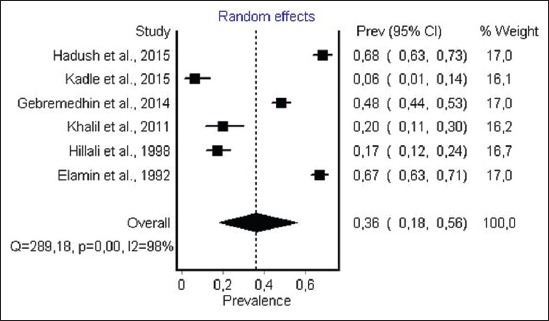
Forest plot of *Toxoplasma gondii* infection prevalence in camel (random-effects model).

For camels, 6 studies from 4 African countries were obtained. Most countries concerned were East African countries: Sudan, Ethiopia, and Somalia. For diagnostic tests, 3 studies used LAT and 3 used DAT. The total number of individual samples was 1621. Prevalence varied from 6.3 to 68.2. The overall estimated prevalence (Figure-6) for toxoplasmosis in camel by random-effect model was 36% (95% CI 18-56%). The result of heterogeneity was also 98.28% (95% CI 97.47-98.81%) for the degree of inconsistency.

#### Prevalence of anti-T. gondii antibody in pig

**Figure-7:**
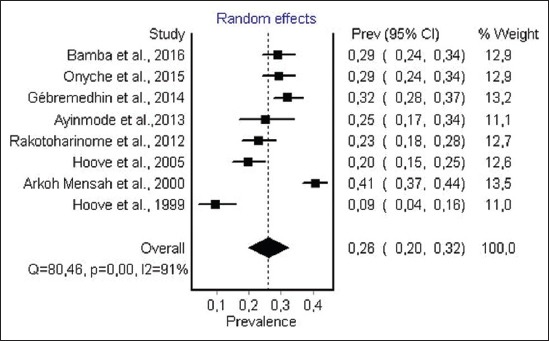
Forest plot of *Toxoplasma gondii* infection prevalence in pig (random-effects model).

Data on *T. gondii* infection in pig were obtained from 8 studies from 6 countries in Africa. 4 studies, performing ELISA, 2 studies, performing MAT and 1 study performing DAT and IFAT respectively. A total number of individual sampled was 2330. Prevalence varied from 9.3 to 40.6. Overall estimated prevalence for anti-*T. gondii* antibody in pig (Figure-7) was 26.0% (95% CI 20.0-32.2). The result of heterogeneity was also 91.3% (95% CI 85.26-94.8) for the degree of inconsistency. Detailed description of each study is given in Figure-7.

#### Prevalence of anti-T. gondii antibody in chicken

**Figure-8:**
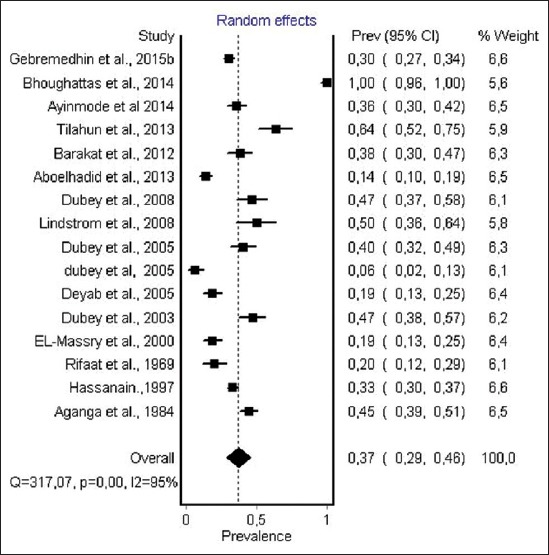
Forest plot of *Toxoplasma gondii* infection prevalence in chicken (random-effects model).

Out of the 16 sero-epidemiological studies from 8 countries in the African continent, 12 studies used MAT, 2 used IHA and 1 study used ELISA and SFT, respectively, for diagnostic of anti-*T. gondii* antibody in chicken. The total number of individual chicken samples for serological testing was 2948. The prevalence of anti-*T. gondii* antibody ranged from 6.3% to 100%. The random effect model gave an overall estimated prevalence (Figure-8) of 37.4% (95% CI 29.2-46.0). The result of heterogeneity was also 95.2% (95% CI 93.6-96.6) for the degree of inconsistency.

### Risk factor

About 18 papers out of 68 selected articles for this systematic review reported statistically significant risk factors for the presence of anti-*T. gondii* antibody in different food animals.

Among sheep and goat, six main risk factors for the presence of anti-*T. gondii* antibody were identified from different studies. It was: Age (Table-1) [49,56,69,86], management farming system (Table-1) [56,75,78], farm location (Table-1) [57,60,69,86], climatic condition (Table-1) [49,74], sex [48,49], and breed (Table-1) [50,78]. Moreover, three of this main risk factors were also identified in cattle namely: Age (Table-1) [40], management system (Table-1) [79], and sex (Table-1) [40].

Among pigs, in addition to age (Table-1) [88]; management system (Table-1) [40,80] and breed [88]; the main risk factor identified was feeding type containing bio products (Table-1) [42].

Otherwise, among chicken, the major risk factor for presence of anti-*T. gondii* were cats density (Table-1) [37] and management system (Table-1) [64].

## Discussion

Toxoplasmosis is one of the most widespread zoonoses in warm-blooded animals. The results of this review allowed us to compare estimates of infection with *T. gondii* and exposure to the parasite in different food animals from Africa. *T. gondii* infection is widespread in some food animals, especially chicken, camel, pig, sheep, and goats which represent the most consumed animal species in Africa for their meat, and there is a wide disparity between the levels of infection in different animal species considered.

The estimated prevalence of anti-*T. gondii* antibody in ruminants was significantly different: Camels, 36% (95% CI 18-56%); sheep, 26.1% (95% CI 17.0-37.0) and goat, 22.9% (95% CI 12.3-36.0%) were the most infected hosts, while the lowest seroprevalence were recorded in cattle 12% (95% CI 8-17%). The highest infection levels are recorded in chickens 37.4% (95% CI 29.2-46.0%), while moderate pooled seroprevalence were obtained in pigs 26% (95% CI 20.0-32.0). However, within each animal species a visible heterogeneity was observed, with a seroprevalence of antibodies ranging from 3.6% to 100% (Table-1) [46,79], as shown in the forest plots (Figures-3-8).

The overall pooled estimate in small ruminants was significant and the infection is more common in sheep which represents the most sensitive species to infection [8]. The highest prevalence were obtained in Ethiopia, 74.80% (Table-1) [74] and Zimbabwe, 68.58% (Table-1) [78]. This result shows the variability of infection rates from one region to another within the same species. In most serological studies from sheep and goats included in the meta-analysis, age is considered an important risk factor, as higher seropositivity is found in older animals (Table-1) [49,56,69,86]. This result is in agreement with the results of studies conducted in France and Iran but in all the world [105-107]. According to many authors, the highest prevalence were reported in farms with epizootic abortions (Table-1) [58,108], while lower seroprevalence was recorded in intensively managed sheep systems (Table-1) [56,78,109]. Toxoplasmosis causes heavy economic losses to sheep industry worldwide and losses are mainly due to abortion and other reproductive failure [110-111]. The ingestion of undercooked meat from infected sheep, especially lamb is considered an important source of infection for humans [112]. Therefore, the estimate demonstrates the risk associated with the consumption of raw products derived from small ruminants in countries where the infection rate is high (Table-1) [50,68]. Usually, raw or undercooked lamb meat is considered a delicacy in some countries and is therefore considered an important source of infection. On the other hand, adult sheep meat is often well cooked, and therefore, probably poses a lower risk of infection to the consumer than lamb meat [112].

In pigs, *T. gondii* infection prevalence ranged from 26.80 to 40.60 excluding one study from Zimbabwe in 1999 reporting a prevalence of 09.60 (Table-1) [89], and lower prevalence rates were recorded in other regions around the world. Thus, prevalence of 28.9% was found in fattening pigs in Serbia [113], 20% in Argentina [114], and 15.6% in Portugal [115]. Poljak *et al*. [116] reported prevalence in pig farms from Canada of 11.6 in 2001, 0% in 2003 and 1.2% in 2004. High infection rate recorded in some African countries may be due to an extensive management system of pigs which is very widespread in Africa. Studies conducted in Ghana, Ethiopia and Zimbabwe have shown that a high prevalence of *T. gondii* was observed in extensively managed pig or backyard scavenging pigs than an intensively managed pig, hence the importance of modern intensive farming systems in reducing the prevalence of *T. gondii* infection in domestic pigs (Table-1) [36,80]. According to Gamble et *al*., the prevalence of *T. gondii* in pigs is also influenced by management systems [117]. In poorly managed non-confinement systems, seroprevalence in pigs was as high as 68% [8]. Moreover, most studies conducted in Ghana, Zimbabwe and Ethiopia revealed that, the age of the animal, the Breed, and the management practices appeared to be the major determinants of prevalence of antibodies against *T. gondii* (Table-1) [40,80,88]. Most pigs acquire *T. gondii* infection postnatally by ingestion of oocysts from contaminated environment or ingestion of infected tissues of animals. Few pigs become infected prenatally by transplacental transmission of the parasite. Raising pigs indoors in confinement has greatly reduced *T. gondii* infection in pigs, but the recent trend of organic farming is likely to increase *T. gondii* infection in pigs [8]. The consumption of pork infected by *T. gondii* is one of the main risk factors for human infection [5,112]. Pork is known as one of the most important sources of T. gondii infection in many countries such as China and USA, most human infections were associated with Pork consumption [3].

The highest estimated prevalence of anti *T. gondii* antibody was record in chickens 37.41% (95% CI 29.20-46.00%) with seroprevalence that ranged from 6.32% to 100% (Table-1) [46,76]. Chickens are considered one of the most important hosts in the epidemiology of *T. gondii* infection because they are an efficient source of infection for cats that excrete the environmentally resistant oocysts and because humans may become infected with this parasite after eating undercooked infected chicken meat [118]. Studies from Tunisia, Ethiopia, and Uganda revealed very high prevalence of anti-*T. gondii* antibody among chicken, not encountered in any African country (Table-1) [46,54,72], suggesting high environmental contamination by oocysts of *T. gondii* excreted by cats in these countries. the prevalence of 24.4% was reported in free-range (FR) chickens from Indonesia, 12.5% in chickens from Italy, 30% in chickens from Poland, and 24.2% in chickens from Vietnam by Dubey *et al*. (Table-1) [71]. In rural areas from Brazil, a prevalence higher than 50% in free ranging chickens was identified, indicating also a widespread contamination of rural environment of that country with *T. gondii* oocysts [119]. Furthermore, the prevalence rates were higher among FR than commercial farm chickens according to many authors (Table-1) [37,64]. Higher seroprevalence particularly in free range chickens (house-reared) refers to the public health importance of chickens as source of zoonotic toxoplasmosis to human (Table-1) [47,64]. In developing sub-Saharan countries, chickens are killed at home or in unsupervised slaughter facilities and the viscera are left for scavengers or are improperly disposed and *T. gondii* infection can be transmitted to human if care is not taken to wash hands thoroughly after cutting meat and during cooking of meat [120].

Results indicate that the estimated prevalence of toxoplasmosis in cattle from Africa is the lowest obtained 12% (95% CI 8-17%, p<0.001) among different food animals. The highest and the lowest prevalence were recorded in Sudan, 32%, and Tanzania, 4%, respectively (Table-1) [67,78]. This overall estimate is higher than the infection rate reported in North of Portugal that was estimated at 7.5% in cattle [121]. In West Indies, a prevalence of 8.4% was reported [122]. In Brazil, the reported sero-prevalence was 49.4% in cattle from a highly endemic area of human toxoplasmosis [123]. Whereas in Malaysia and Vietnam, lower seroprevalence of 7.9% and 10.5% were, respectively, reported in cattle [124,125]. High prevalence of toxoplasmosis of cattle in some areas may be due to the following factors: Humid and temperate climate; the absence of routine treatment against feline toxoplasmosis, considerable cat abundance and last but not least exposure to cats and their oocysts. Several epidemiological studies have mentioned that the consumption of raw or undercooked beef could be considered as a risk for *T. gondii* infection in humans [126]. But according to Kijlstra and Jongert [112] and Dubey and Jones [3] transmission from cattle is not important for human infection. Given the low level of infection in cattle from Africa, we can assume that the risk for *T. gondii* infection in humans from beefs is low as compared to other hosts of *T. gondii*. Among ruminants, camels are the most infected species by *T. gondii*, 36% (95% CI 18-56%). *T. gondii* infection rate in Africa ranged from 17% to 68% and the highest rates were obtained in Sudan (Table-1) [97]. A higher prevalence has been reported from Turkey (90.9%) [127], while lower seroprevalence was recorded earlier from Iran 3.12% [128] and Saudi Arabia 6.5% [129].

Overall, the variation of seroprevalence of *T. gondii* infection among different species might be due to the difference in density of cats and wild felids around farm, climatic conditions [130], farming and management practices [3], sample size, cutoff titer, duration of studies, and sensitivity difference in the serological tests employed. According to Guo *et al*. [131], the heterogeneity in prevalence could also be related to the presence of risk factors including farm type, feeding practices, presence of cats, rodent control and bird control methods, farm management, carcasses handling and disposal, and water source and quality. Moreover, studies carried out in distinct countries and various climatic conditions affect the results that could be another reason for this heterogeneity.

Results from some studies showed significant relation between animal age and *T. gondii* infection among all hosts. It shows a higher prevalence in adults animals than young which may be resulted from more exposure during animal growth. Animals acquire *Toxoplasma* infection merely via ingestion of oocyst and when prevalence is considerably high. There is a widespread oocyst contamination of the environment because of fecal contamination of soil and groundwater either by domestic or feral cats. Understanding prevalence rate of animal toxoplasmosis will help us to estimate the rate of human toxoplasmosis and it can be a good indicator of environment and final host contamination [107]. This point is extremely important to mention that it is not easy to consider prevention and control program without enough information about prevalence of toxoplasmosis in animal since they are a major source of transmission to human.

Given the vital role of animals in the transmission of *T. gondii* to humans via their products (meat and milk) and the prominent role of cats in disseminating and contamination of the environment by oocysts [1], more emphasis should be placed on the prevention of animal toxoplasmosis in Africa.

Caution is warranted in the interpretation of results of *T. gondii* prevalence in camel. Regarding such species, the prevalence data used in this study were analyzed based on a limited number of national studies, and nationwide surveys are not available in these meat animals, which resulted in a wide 95% CI of the estimated prevalence.

## Conclusion

This systematic review was performed to evaluate the prevalence of *T. gondii* infection among sheep, goat, cattle, pig, camel, and chicken which represent the most consumed food animal species in different African countries. The Random-effects meta-analysis approach in this current study provided an estimate of *T. gondii* prevalence in various meat animals with an increased level of precision. The widespread prevalence of *T. gondii* in sheep, chicken, camel, pig, and goats indicates a food safety concern in different African countries, especially countries where the infection is more important. Other studies are required for a better and continual evaluation of the occurrence of this zoonotic infection.

## Authors’ Contributions

The study was conceptualized and protocols were carried out by YA. ABNT and PS were involved in the database search, data extraction, statistical analysis, and manuscript written. CA and EY Studied titles and abstract of all the articles and retrieved data. Quality assessment of each study was completed independently by YGH and IY. MNA and SF oversaw data collection and analysis of statistical results. All authors have read and approved the content.
